# Cloning and Characterization of a P2X Receptor Expressed in the Central Nervous System of *Lymnaea stagnalis*

**DOI:** 10.1371/journal.pone.0050487

**Published:** 2012-11-29

**Authors:** Selvan Bavan, Volko A. Straub, Tania E. Webb, Steven J. Ennion

**Affiliations:** 1 Department of Cell Physiology and Pharmacology, University of Leicester, Leicester, United Kingdom; 2 Leicester School of Pharmacy, De Montfort University, Leicester, United Kingdom; University of Waterloo, Canada

## Abstract

P2X receptors are membrane ion channels gated by extracellular ATP. Mammals possess seven distinct P2X subtypes (P2X1-7) that have important functions in a wide array of physiological processes including roles in the central nervous system (CNS) where they have been linked to modulation of neurotransmitter release. We report here the cloning and functional characterization of a P2X receptor from the mollusc *Lymnaea stagnalis*. This model organism has a relatively simple CNS consisting of large readily identifiable neurones, a feature which together with a well characterized neuronal circuitry for important physiological processes such as feeding and respiration makes it an attractive potential model to examine P2X function. Using CODEHOP PCR we identified a single P2X receptor (*Lym*P2X) in *Lymnaea* CNS which was subsequently cloned by RT-PCR. When heterologously expressed in *Xenopus* oocytes, *Lym*P2X exhibited ATP evoked inward currents (EC_50_ 6.2 µM) which decayed during the continued presence of agonist. UTP and ADP did not activate the receptor whereas αβmeATP was a weak agonist. BzATP was a partial agonist with an EC_50_ of 2.4 µM and a maximal response 33% smaller than that of ATP. The general P2 receptor antagonists PPADS and suramin both inhibited *Lym*P2X currents with IC_50_ values of 8.1 and 27.4 µM respectively. *Lym*P2X is inhibited by acidic pH whereas Zn^2+^ and Cu^2+^ ions exhibited a biphasic effect, potentiating currents up to 100 µM and inhibiting at higher concentrations. Quantitative RT-PCR and *in situ* hybridization detected expression of *Lym*P2X mRNA in neurones of all CNS ganglia suggesting this ion channel may have widespread roles in *Lymnaea* CNS function.

## Introduction

In addition to its role as the primary energy source within cells, adenosine 5′-triphosphate (ATP) also acts as a signalling molecule and is thought to be one of the earliest transmitters to have appeared during the evolution of eukaryotes [1]. ATP initiates intracellular signalling pathways by activating either fast acting P2X ion channels or slower acting P2Y G-protein coupled receptors. P2X channels form as homo or heteromeric trimers [2] with each individual subunit consisting of intracellular amino and carboxy termini, two transmembrane domains and a large extracellular loop containing five disulphide bonds [3] and the ATP binding sites which form across neighbouring subunits [4], [5]. Mammalian species possess seven P2X receptor subtypes (P2X1-7), each encoded by a separate gene, which form non-selective cation channels in the plasma membrane upon gating by extracellular ATP [6], [7]. P2X receptors have also been cloned from a range of other vertebrate species, the most significant of which being zebrafish P2X4 [8] since this was subsequently crystallised to allow structural determination of both apo and agonist bound states [5], [9]. The first P2X receptor identified in an invertebrate organism was from the blood fluke *Schistosoma mansoni* [10] and subsequently P2X receptors from more primitive organisms such as the amoeba *Dictyostelium discoideum* [11], [12], the green algae *Ostreococcus tauri* [13], the choanoflagellate *Monosiga brevicollis* [13] and three species of basal fungi [14], [15] were also described as well as additional invertebrate P2X receptors from the tick *Boophilus microplus* [16] and the tardigrade *Hypsibius dujardini* [17]. Interestingly, the five P2X receptors present in *Dictyostelium discoideum* are localized to the contractile vacuole, an intracellular organelle involved in osmoregulation and Ca^2+^ release [11], [12], [18].

P2X mediated signalling plays a fundamental role in a wide array of physiological processes including smooth muscle contraction, inflammation, bone formation and platelet aggregation [19]. P2X receptors are also widely distributed in the central nervous system (CNS) where they are involved in processes such as synaptic transmission [7], [20], long term potentiation [21] and taste sensation [22]. The roles played by P2X receptors in CNS function are often complex and difficult to study. One potential strategy which could be of use in gaining a better understanding of these roles could be to study P2X receptor function in the CNS of a simple model organism. The pond snail *Lymnaea stagnalis* has a relatively simple CNS containing ∼20,000 readily identifiable neurons [23] and has historically proved to be an extremely useful and accessible model to study fundamental aspects of CNS function such as synaptic plasticity [24] and associative memory [25]. Furthermore, the neuronal pathways underlying complex physiological processes such as feeding and respiration have been elucidated in this organism [26]–[28], making it an attractive model for investigating neural networks. The demonstration that ATP is released from *Lymnaea stagnalis* CNS ganglia [29] suggests the presence of a purinergic signalling system and it is therefore possible that *Lymnaea* could potentially be developed as a simple model system to study P2X receptor function in the CNS.

With this potential benefit in mind, this study aimed to determine whether P2X receptors are expressed in the CNS of *Lymnaea stagnalis*. In the absence of any prior EST or genomic sequence data for a potential *Lymnaea* P2X receptor, we utilised CODEHOP PCR to identify a P2X receptor expressed in *Lymnaea stagnalis* CNS. The cDNA for this receptor was subsequently cloned and heterologously expressed in *Xenopus* oocytes to confirm that it encoded an ATP gated ion channel and to determine its pharmacological characteristics.

## Materials and Methods

### Cloning of the *Lymnaea Stagnalis* P2X Receptor

P2X CODEHOP PCR primers were designed using the CODEHOP algorithm [30] with input blocks generated from predicted extracellular region amino acid sequences (from the end of transmembrane domain 1 to the start of transmembrane domain 2) of the mammalian P2X1-7 and available invertebrate P2X receptors using the BlockS WWW server (Fred Hutchinson Cancer Research Centre).

Total RNA was isolated from dissected *Lymnaea* CNS using a scaled down (500 µl total volume) version of the Chomczynski method [31] and 5 µg used in a first strand cDNA reaction using Oligo dT_(17)_ primer and Bioscript reverse transcriptase according to the manufacturer’s instructions (Bioline, U.K.). First strand cDNA (0.5 µl) was used directly as template in a PCR reaction containing 200 µM each dNTP, 1.5 mM MgCl_2_, 25 pmoles each of CODEHOP primer pair 1 (Table 1), 1× NH4–based reaction Buffer (Bioline) and 2.5 Units BIOTAQ DNA polymerase (Bioline) added after a hot start of 94°C for 2 minutes. Thermal cycling consisted of 40 repetitions of 94°C for 30 seconds 54°C for 30 seconds, and 72°C for 40 seconds. This initial CODEHOP PCR reaction was subsequently used as template (0.5 µl) in a second nested PCR reaction using the same reaction conditions as the initial amplification and primer pair 2 (Table 1). 5′RACE was conducted on *Lymnaea* CNS using a FirstChoice® RLM-RACE kit according to the manufacturer’s instructions (Ambion, U.S.A.) with primer pairs 3 and 4 (Table 1). 3′ sequence of the *Lym*P2X gene was obtained by conducting PCR on a *Lymnaea* cDNA Lambda ZAP® II library which was kindly provided by Dr Sergei Korneev, University of Sussex. Reactions consisted of 1 µl cDNA library DNA, 200 µM each dNTP, 1.5 mM MgCl_2_, 25 pmoles each of primer pair 5 (Table 1), 1× NH_4_–based reaction Buffer (Bioline) and 2.5 Units BIOTAQ DNA polymerase (Bioline) with thermal cycling of 40 repetitions of 94°C for 30 seconds 50°C for 30 seconds, and 72°C for 1 minute. Amplicons of the expected size obtained from CODEHOP, 5′RACE and cDNA library PCR were excised from agarose gels, purified using a QIAquick gel purification kit (Qiagen) and directly sequenced (University of Leicester automated sequencing service). To generate full length coding sequence clones, RT-PCR was performed on *Lymnaea* CNS cDNA using primer pairs 6 and 7 (Table 1). A consensus Kozak sequence was incorporated into the forward primer of each pair. PCR reactions consisted of 0.25 µl cDNA, 6.25 pmoles each primer, 200 µM each dNTP, 2 mM MgCl_2_, 1 X Optibuffer (Bioline) and 1.6 Units Bio-X-Act DNA polymerase (Bioline). Thermal cycling consisted of 30 repetitions of 94°C for 30 s, 50°C for 1 min, and 72°C for 1.5 min. Amplicons were visualised on a 0.8% agarose gel, purified using a QIAquick gel extraction kit (Qiagen) and cloned into a pcDNA3.1 based plasmid vector by TA-cloning. Independent clones were subsequently sequenced on both strands.

**Table 1:** Oligonucleotide primers.

	Name	Forward primer (5′-3′)	Name	Reverse primer (5′-3′)
1	BlockA4For	TGTTGGGTCTTGGTCTACAAGAARGGNTAYCA	BlockFRev	CGATGTTGCCGAACTGGAANAYNGGRCA
2	BlockA2For	CCGCCGTGACCACCAARRTNAARGG	WCP1REV	TGGCATTTTGTCGTTCTCGRNNGGRCACCA
3	5′ RACE Outer*	GCTGATGGCGATGAATGAACACTG	LymRACEout	CCTTTCAGGCGTTCACAATA
4	5′ RACE Inner*	CGCGGATCCGAACACTGCGTTTGCTGGCTTTGATG	LymRACEin	CTTTGGTGGTCACAGCACTT
5	LymForB	TGAAAGGGACTATAATGCCA	ZAPREV2	CCTCACTAAAGGGAACAAAA
6	LymKOZMTTfor	GCCGCCACCATGAATTTCAGAAATATTGATTGG	LymFULLrevin	AGGCTCGGGACTTATCAA
7	LymKOZMADfor	GCCGCCACCATGGCTGACCCAAAACACTG	LymFULLrevin	AGGCTCGGGACTTATCAA
8	LymP2Xfor	GGGATCGTCTTCGTGGTGA	LymP2Xrev	TGTCCTGAGGCGACTCTTCTT
9	β-tubulinfor	GAAATAGCACCGCCATCC	β-tubulinrev	CGCCTCTGTGAACTCCATCT
10	LyT7FORinsit	GAAATTAATACGACTCACTATAGGGACTATAATGCCAGGAGG	LyminsituREV	CCTTCAACAGATAGAGCACGATG
11	LyminsituFOR	GGGACTATAATGCCAGGAGG	LyT7REVinsit	GAAATTAATACGACTCACTATAGGGCC TTCAACAGATAGAGCACG

Primer pairs 1−7 = cloning of LymP2X, * = Primers part of 5′RACE kit. 8−9 = primers used for RT-PCR and QRT-PCR. 10−11 = Primers used to generate templates for synthesis of sense (10) and antisense (11) cRNA probes used for *in situ* hybridization.

### Oocyte Preparation

Plasmid DNA encoding *Lym*P2X was digested with *MluI* to linearize and used as template to transcribe sense strand cRNA using a T7 mMessage mMachine™ kit (Ambion, U.S.A.) according to the manufacturer’s instructions. Manually defolliculated stage V-VI *Xenopus* oocytes were injected with 5 ng of cRNA in a volume of 50 nl using an Inject +Matic micro injector (J.Alejandro Gaby, Genève) and were stored at 18°C in ND96 buffer (96 mM NaCl, 2 mM KCl, 1.8 mM CaCl_2_, 1 mM MgCl_2_, 5 mM sodium pyruvate, and 5 mM HEPES, pH 7.5) before recordings 3–6 days later.

### Two-electrode Voltage Clamp

Two-electrode voltage clamp recordings were made from oocytes using a Turbo TEC 10 C amplifier (NPI Electronic Instruments, Germany) with a Digidata 1200 analogue to digital converter (Axon Instruments U.S.A.) and WinWCP acquisition software (Dr J. Dempster University of Strathclyde, Scotland). Microelectrodes were pulled with a resistance of 0.2 MΩ and filled with 3 M KCl. The external recording solution consisted of ND96 buffer containing 1.8 mM BaCl_2_ instead of 1.8 mM CaCl_2_ to prevent the activation of endogenous oocyte calcium-activated chloride channels. Oocytes were clamped at a holding potential of –60 mV. Agonists, ATP (Mg^2+^ salt), 2′,3′-O-4-Benzoylbenzoyl ATP (BzATP), α,β-methylene-adenosine 5′-triphosphate (αβmeATP), ADP and UTP (Sigma, Poole, U.K.) were applied from a U-tube perfusion system, whereas pyridoxal-phosphate-6-azophenyl-2',4'-disulfonic acid (PPADS), suramin, zinc, copper and altered pH solutions were bath-perfused as well as being present at the same concentration in the U-tube agonist solution. Concentration-response curves were constructed using a 5-minute recovery period between agonist applications. Test concentrations of agonist were applied in a randomised order for different oocytes and data points normalized to two “bracketing” applications of 10 µM ATP (one 5 minutes preceding and one 5 minutes following the test concentration).

### Quantitative Real-Time PCR

*Lymnaea Stagnalis* CNS were dissected into the seven ganglia components: buccal, cerebral, pedal, pleural, left parietal, right parietal and visceral ganglia. Total RNA was isolated from each ganglion using the RNeasy Fibrous Tissue Mini Kit (Qiagen) and genomic DNA contamination removed by DNAse digestion. RNA was quantified using a Nanodrop spectrophotometer and ganglia samples diluted to 1 ng/µl. RNA from each ganglia was reverse transcribed to first strand cDNA using oligo-dT primer and Bioscript reverse transcriptase (Bioline) according to the manufacturer’s instructions. Negative controls consisted of a no reverse transcriptase reaction where exactly the same procedures were followed with the omission of reverse transcriptase in the cDNA reaction. For quantitative PCR cDNA for each ganglia sample was synthesised from 300 pg of RNA and run in a 25 µl PCR reaction with 1 x SYBR Green mix containing heat-activated Taq DNA polymerase, reaction buffer, dNTPs, 3 mM MgCl_2_, internal reference dye, stabilisers and SYBR Green I (SYBR Green Jump Start Taq ReadyMix for High Throughput qPCR (Sigma)) and 75 nM gene specific primers (either primer pair 8 for *Lym*P2X or primer pair 9 for β-tubulin). The expected PCR product amplicon sizes were 229 bp for the *Lym*P2X primer pair and 128 bp for the β-tubulin primer pair. Thermal cycling (MJ-Research Thermo Cycler RT-PCR machine) consisted of 94 °C for 2 min followed by 35 cycles of 94 °C for 15 s, 55 °C for 1 min and 72 °C for 30 s followed by an incubation at 72°C for 10 min. C_t_ values were taken at a threshold of 0.01 units of fluorescence. *Lym*P2X expression in each ganglia was quantified using the comparative C(T) method [32] where C_t_(*Lym*P2X) = number of PCR cycles for *Lym*P2X primers to reach 0.01 threshold, C_t_(β*-*tubulin) = number of PCR cycles for β*-*tubulin primers to reach 0.01 threshold, ΔC_t_ = C_t_(*Lym*P2X)−C_t_(β-tubulin), ΔΔC_t_ = ΔC_t(X)_− ΔC_t(Z)_ (where ΔC_t(X)_ = ganglion sample ΔC_t_ and ΔC_t(Z)_ = ganglion sample with the highest ΔC_t_ and therefore lowest relative expression level). The relative quantity of *Lym*P2X in each ganglia sample was then expressed as a multiple of ganglia sample Z: *Lym*P2X^N^ = 2^−ΔΔCt^_._ Melting curves carried out at 1 degree intervals between 50–95 degrees were used to check the purity of PCR products. Completed PCR reactions were also separated on a 1.75% agarose gel containing ethidium bromide in order to verify the correct size product (∼230 bp for *Lym*P2X and ∼130 bp for β-tubulin). Primer amplification efficiencies were previously determined by conducting qRT-PCR reactions over a range of input RNA concentrations (1 ng, 0.3 ng, 0.1 ng, 0.03 ng, and 0.01 ng). A graph of ΔCt (Ct(*Lym*P2X)−Ct(β-tubulin)) versus log cRNA gave a slope of −0.004±0.08 demonstrating that the *Lym*P2X and β-tubulin primer pairs had comparable amplification efficiencies (105.4% and 93.4% respectively).

### *In situ* Hybridization

Digoxigenin-labelled sense and antisense RNA probes were synthesised using a DIG RNA Labelling Kit (Roche, UK) according to the manufacturer’s instructions. DNA templates for RNA synthesis were prepared by PCR on the *Lym*P2X plasmid using primer pairs 10 (sense probe) and 11 (antisense probe) (Table 1) so as to introduce a T7 polymerase promoter site at the appropriate position. Each primer pair generated a PCR product of 728 bp. Transcription of both sense and antisense RNA from these PCR templates gave transcripts of 703 bases corresponding to nucleotide positions 444–1146 in the *Lym*P2X sequence (GenBank JX524180). Dissected *Lymnaea* CNS were fixed in 4% paraformaldehyde for 4 hours at 4°C and dehydrated in 25%, 40%, 60%, and 70% ethanol (10 min each) before being processed in a standard automated histological wax embedding machine. Sections at a thickness of 5 µm were cut on a sledge microtome, mounted on Plus™ slides (VWR, UK) and dried overnight at 37°C before storage at room temperature. Prior to hybridization, sections were dewaxed in xylene and rehydrated through graded alcohols in PBS (100% twice, 90%, 70%, and 40% for 2 min each) before post fixing in 4% paraformaldehyde in DEPC-treated PBS for 20 minutes. Slides were then washed twice for 15 min in PBS with 0.1% DEPC at room temperature and equilibrated in 5 X SSC before prehybridization in 50% formamide, 5 X SSC, 40 µg/ml salmon sperm DNA at 58°C for 2 hours. Hybridization was carried out in 50% formamide, 5 X SSC, 40 µg/ml salmon sperm DNA and 400 ng/ml of DIG-labelled cRNA probe at 58°C for 20 hours. Post-hybridization washes consisted of a 30 min wash in 2 X SSC at room temperature, a 1 hour wash in 2 X SSC at 65°C, and a 1 hour wash in 0.1 X SSC at 65°C. This was followed by a 30 min equilibration in blocking buffer, and a 2 hour incubation with alkaline phosphatase conjugated anti-DIG antibody diluted (1∶2000) in blocking buffer. Two 15 min washes were then carried out with washing buffer (Roche). Slides were equilibrated in detection buffer for 5 min before addition of substrate solution (45 µl NBT and 35 µl BCIP per 10 ml of detection buffer). Reactions were stopped in distilled water, followed by a one hour incubation in 95% ethanol. Slides were then washed twice in distilled water for 15 min, followed by dehydration and mounting in DPX (Sigma).

### Data Analysis

Data are presented as means ± s.e.m. Differences between means were assessed by Student’s t-test. Concentration-response data were fitted with the equation *Y* = ((*X*)*^H^*·*M*)/((*X*)*^H^*+(EC_50_)*^H^*), where *Y* is response, *X* is agonist concentration, *H* is the Hill coefficient, *M* is maximum response, and EC_50_ is the concentration of agonist evoking 50% of the maximum response. Concentration-response curves, EC_50_ values and Hill coefficients were obtained using GraphPad Prism software (La Jolla, USA).

**Figure 1:**
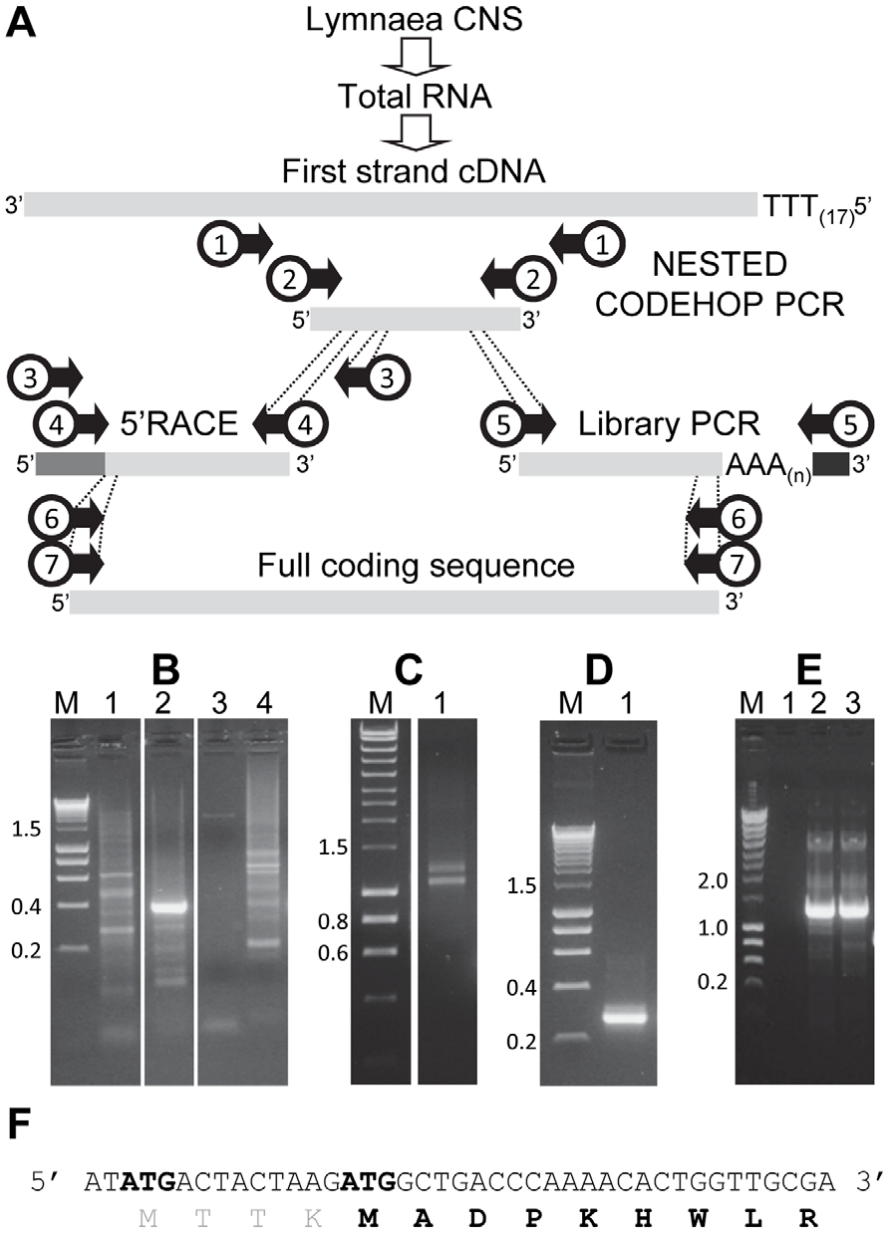
Overview of *Lym*P2X cloning strategy. (A) Nested CODEHOP PCR was performed on cDNA prepared from *Lymnaea* CNS to generate a *Lym*P2X gene amplification product. The sequence of this internal product could then be utilised to design gene specific primers to facilitate amplification of the 5′ end of the gene by 5′RACE and the 3′ end of the gene by PCR on a *Lymnaea* cDNA library. Once the 5′ and 3′ sequence of the gene had been determined, further gene specific primers could then be designed to amplify the full length *Lym*P2X coding sequence from *Lymnaea* cDNA. Numbered arrows indicate primer pairs listed in Table 1. (B) Agarose gel showing separation of CODEHOP PCR products using primer pair 1 with cDNA as template (lane 1), primer pair 2 with PCR reaction 1 as template (lane 2) and control reactions using PCR reaction 1 as template with only the forward primer of primer pair 2 (lane 3) or only the reverse primer of primer pair 2 (lane 4). (C) Amplification of the 3′ end of *Lym*P2X by PCR on a *Lymnaea* cDNA library using primer pair 5 (lane 1). (D) 5′RACE PCR using primer pair 4. (E) Amplification of the full length *Lym*P2X coding sequence using primer pairs 6 (lane 2) or 7 (lane 3). Lane 1 shows no template negative control. M indicates molecular mass ladder (size in kb). (F) Nucleotide and predicted amino acid sequence of the 5′ end of the *Lym*P2X transcript. The two potential start methionines are indicated in bold.

**Figure 2:**
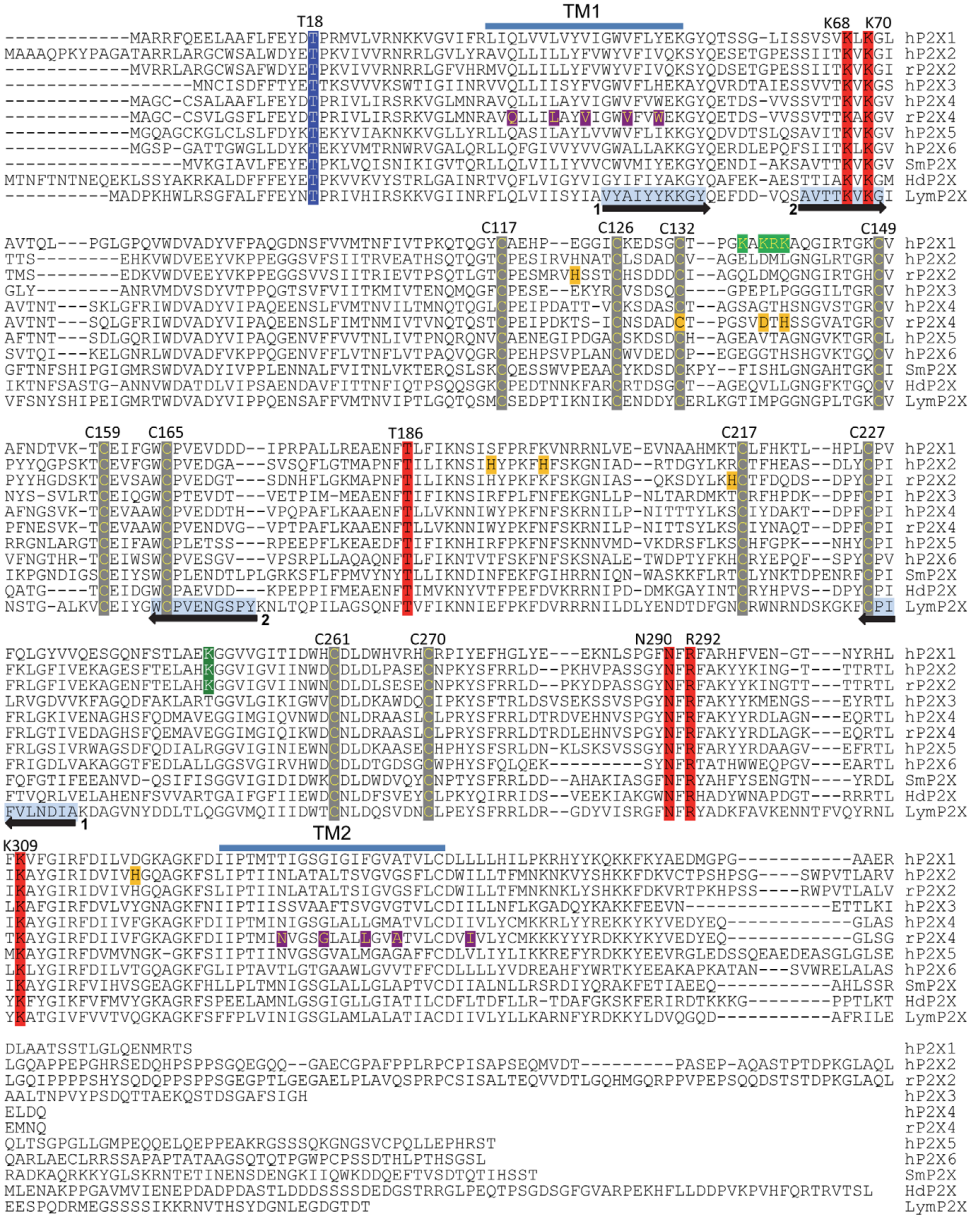
Alignment of the predicted *Lym*P2X amino acid sequence with mammalian and lower organism P2X receptors. The *Lym*P2X amino acid sequence (LymP2X) was aligned with human P2X1-6 (P51575, Q9UBL9, P56373, Q99571, Q93086, O15547), rat P2X2 (P49653), rat P2X4 (P51577), *Schistosoma mansoni* P2X (smP2X) (Q65A65) and *Hypsibius dujardini* P2X (HdP2X) (ACL14328) using CLUSTALW software. Equivalent residues in the two transmembrane domains (TM1 and TM2) of the zP2X4 crystal structure [9] are indicated as horizontal blue bars. Highlighted conserved residues are numbered according to the hP2X1 sequence. Residues involved in ATP binding [5] are highlighted in red. Ten conserved cysteine residues which form five disulphide bonds [3] are in grey with yellow text and the consensus protein kinase C phosphorylation site (T18) in blue. Black arrows with light blue shaded amino acid sequence indicate the locations of CODHOP PCR primer pairs 1 and 2 used to identify *Lym*P2X (Table 1). Rat P2X4 residues thought to be involved in the interaction with ivermectin (Q36, L40, V43, V47 W50, N338, G342, L346, A349 and I356) [59] are highlighted in purple with yellow text. Residues shown to be involved in the actions of metal ions are shaded orange where human P2X2 H204, H209 and H330 control access of zinc to its binding site [55], H120 and H213 in rat P2X2 are involved in the formation of an intersubunit zinc binding site [52]–[54] and D138, H140 and C132 in rat P2X4 are involved in the inhibitory modulation by metal ions [56]. A cluster of four positively charged residues thought to be involved in the actions of suramin and NF449 at hP2X1 (K136, K138, R139 and K140) [60], [61] are shaded light green with yellow text whilst a lysine residue thought to be involved in PPADS action at P2X1 and P2X2 [62] is shaded dark green with white text (K249).

## Results

### CODEHOP PCR to Identify a P2X Receptor Expressed in *Lymnaea* CNS

In the absence of any sequence data for a *Lymnaea* P2X receptor, we utilised the CODEHOP PCR method on cDNA from *Lymnaea* CNS in an attempt to generate internal amplification products from potential P2X genes. A range of consensus P2X based CODEHOP primers were designed and tested according to standard recommendations [30] (see materials and methods). However, none of these individual primer pair combinations were successful in amplifying a clear product of the expected size (data not shown) in standard CODEHOP reactions. We therefore next conducted a nested PCR strategy whereby an aliquot of an initial standard CODEHOP PCR reaction was used as template in a second reaction using a different CODEHOP primer pair predicted to be internal to the first pair. Using this strategy primer pair 2 (Table 1) produced a clear PCR amplicon of 352 bp (Fig. 1B lane 2). Direct DNA sequencing of this PCR product revealed a predicted translated amino acid sequence with high similarity to the equivalent region in mammalian P2X receptors and enabled us to design gene specific primers to facilitate PCR amplification of the 5′ and 3′ ends of the gene. A λ ZAP® II *Lymnaea* CNS cDNA library was subsequently used as template for PCR reactions using vector and gene specific primers. This allowed us to amplify the 3′ end of the gene using primer pair 5 (table 1) which yielded a doublet band at ∼1150 bp (Fig. 1C). Direct DNA sequencing of these two PCR products revealed that the smaller of the two bands corresponded to a P2X clone with overlapping sequence to the CODEHOP PCR product. The larger of the two bands appeared to be a non-specific amplification and was not pursued further. Several 5′end PCR products were also amplified from the library using a reverse gene specific primer and vector specific forward primer but in each case the clones obtained appeared to be truncated and no ATG start site within the coding sequence could be identified. We therefore conducted 5′RACE PCR on *Lymnaea* cDNA and obtained a single 263 bp amplicon (Fig. 1D). Direct sequencing of this 5′RACE product revealed an ATG start codon in a position equivalent to mammalian P2X receptor sequences and a short 5′untranslated region of 14 nucleotides. A second potential in frame ATG codon was also present 2 nucleotides from the cap site (Fig. 1F). Whilst it is unlikely that this site is utilised *in vivo*, a full length coding sequence clone for each start site was generated by RT-PCR on *Lymnaea* cDNA using primer pairs 6 and 7 (Table 1) generating amplicons of ∼1400 bp (Fig. 1E). Clones from independent PCR reactions were sequenced on both strands and the full length cDNA sequence has been deposited in the GenBank database (Accession number: JX524180).

**Figure 3:**
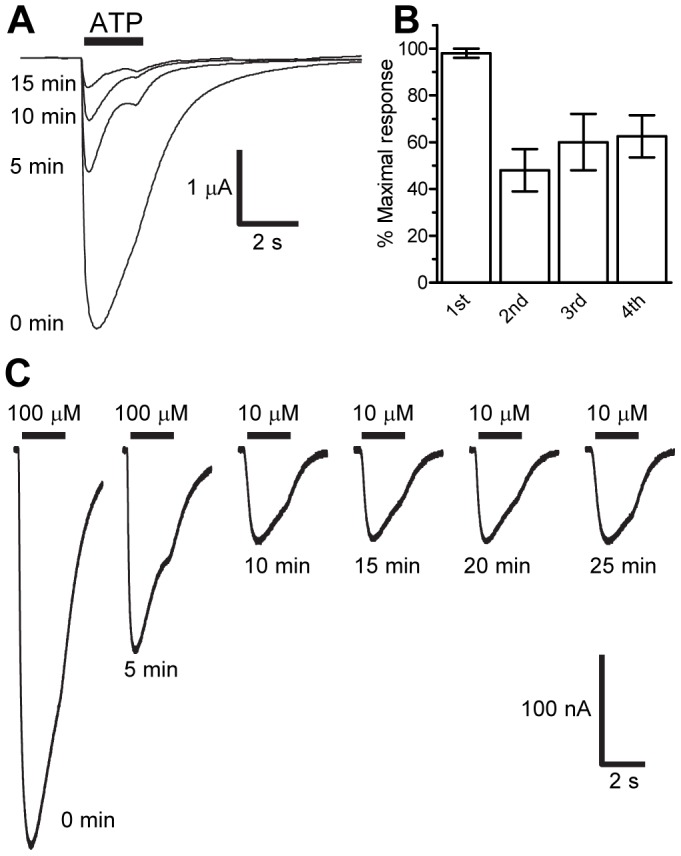
The *Lym*P2X gene encodes an ATP gated ion channel. Membrane currents were recorded by two-electrode voltage clamp in *Xenopus* oocytes expressing *Lym*P2X receptors. (A) Example sequential current traces in response to a 2 second application (solid black bar) of 100 µM ATP showed a marked run down in peak current amplitude with a 5 minute recovery period between applications. (B) Sequential application of ATP (100 µM for 2 seconds) with a 15 minute recovery period between applications showed no rundown in amplitude after the second ATP application (n = 5). (C) A 5 minute recovery period between sequential application of 10 µM ATP (black bars) resulted in reproducible responses after two initial applications of 100 µM ATP.

### Sequence Analysis of *Lym*P2X

The coding sequence of *Lym*P2X encodes 435 amino acids with predicted intracellular amino and carboxy termini, two transmembrane domains and a large extracellular loop. The sequence displays several highly conserved residues typical of the P2X receptor family including 10 cysteine residues that form five disulphide bonds within the extracellular loop of vertebrate P2X receptors [3], a consensus protein kinase C phosphorylation site in the amino terminus, and positively charged and aromatic residues that form the ATP binding site (Fig. 2). The percentage amino acid sequence identity between *Lym*P2X and human P2X1-7 sequences range from 31.0–45.9%, with highest similarity to P2X_4_ and lowest similarity to P2X7.

**Figure 4:**
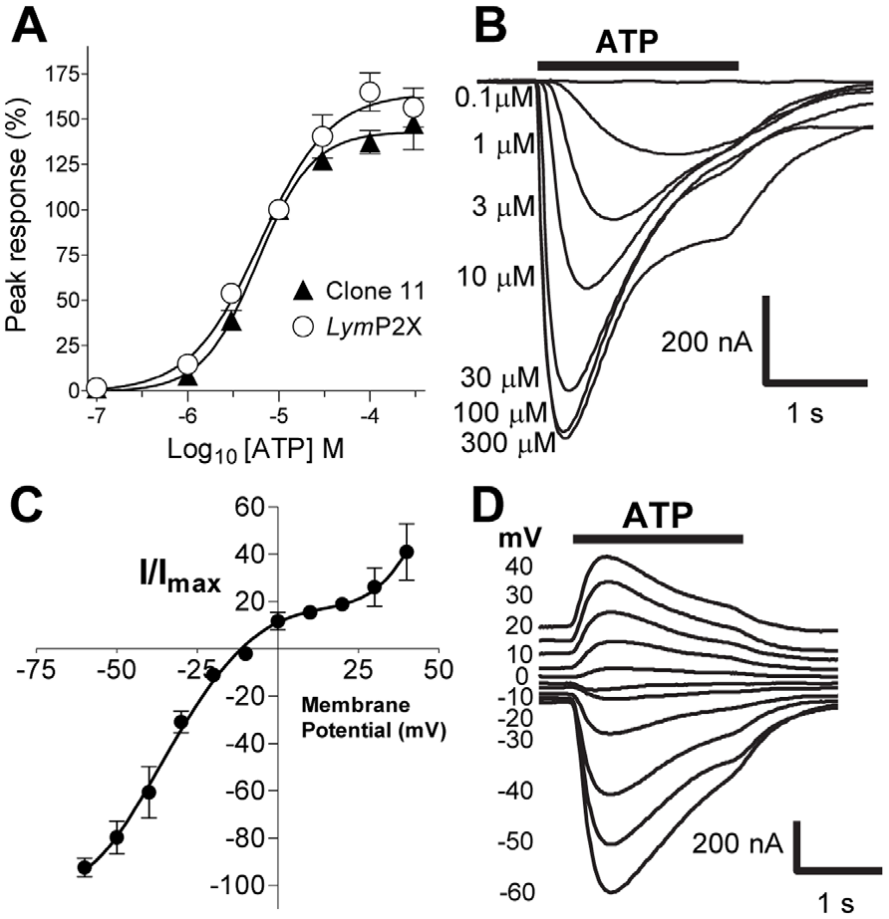
Properties of ATP evoked currents. (A) Concentration response curves for ATP in *Lym*P2X expressing oocytes. Mean peak currents (± s.e.m) were normalized to responses evoked by 10 µM ATP, EC_50_ = 6.2 µM for *Lym*P2X (n = 10 oocytes) and 5.8 µM for clone 11 (n = 5). B) Example *Lym*P2X current traces in response to different concentrations of ATP (black bar). (C) Current voltage relationship of *Lym*P2X. The reversal potential of ATP mediated currents was determined by recording ATP (10 µM, indicated by bar) induced currents at holding potentials ranging from –60 mV to +40 mV with a 5 minute interval between applications. Currents obtained in different oocytes were expressed as a negative percentage of the maximum current for each individual cell (n = 7). D. Example currents for the plot depicted in C.

### *Lym*P2X is an ATP Gated Ion Channel

Application of 100 µM ATP to *Xenopus* oocytes previously injected with *Lym*P2X cRNA evoked an inward current that decayed in amplitude during the continued presence of agonist (Fig. 3A). Water injected control oocytes produced no membrane currents upon ATP application (data not shown). The decay in *Lym*P2X current during the continued presence of agonist showed a T_50_ of 884.32±49.3 ms and could be fitted by a single exponential with a time constant of 1.5±0.2 s whilst the time taken for currents to rise from 10–90% peak was 207.7±20.7 ms (n = 10). These relatively slow current kinetics of *Lym*P2X are similar to ATP-evoked currents of the mammalian P2X_4_ subtype [33].

**Figure 5:**
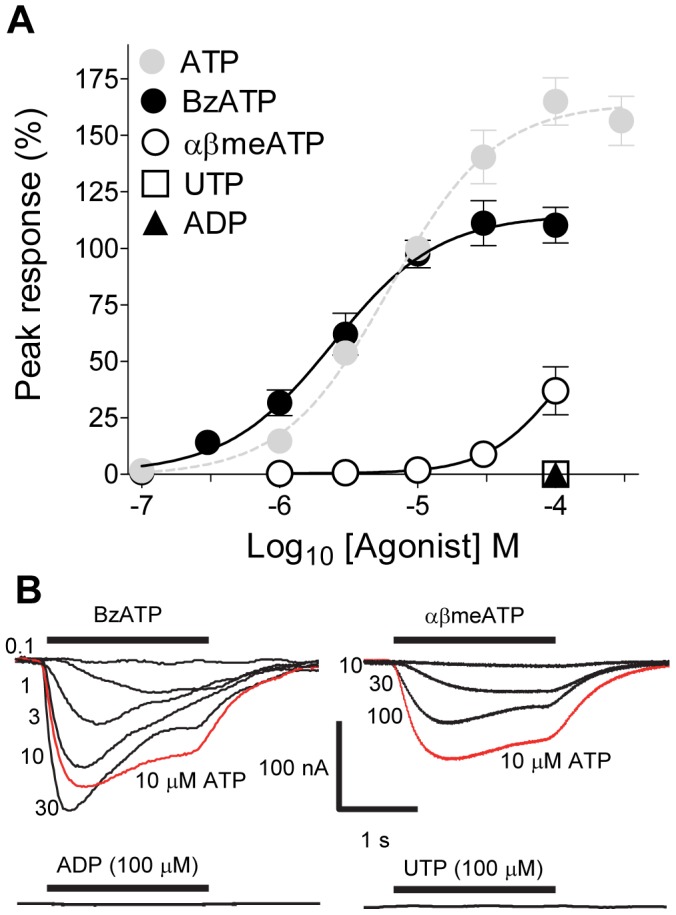
BzATP (2′, 3′-O-(4-Benzoylbenzoyl ATP)) is a partial agonist and αβmeATP a weak agonist at *Lym*P2X. (A) Concentration response curves for BzATP (EC_50_ of 2.4 µM, n = 5−10) and αβmeATP (n = 6). Mean peak currents (± s.e.m) are normalized to responses evoked by 10 µM ATP. The concentration response curve for ATP is also plotted as grey dashed line for comparison. (B) Representative current traces for data plotted in A) (agonist concentrations in µM, 10 µM ATP traces in red). Neither UTP nor ADP (hexokinase treated) evoked membrane currents at *Lym*P2X.

With a 5 minute recovery period between sequential applications of 100 µM ATP, *Lym*P2X currents showed a marked run-down in peak amplitude (Fig. 3A). When the recovery period was increased to 15 minutes, sequential responses after the first application were more consistent showing that 5 minutes was insufficient for full recovery from desensitisation (Fig. 3B). Using a lower concentration of ATP (10 µM) with a 5 min recovery interval also gave consistent responses following two initial applications of 100 µM ATP (Fig. 3C). As a 15 minute recovery interval was impractical for pharmacological characterization of the receptor (*Xenopus* oocytes are typically viable for ∼1 hour under recording conditions), 10 µM ATP was subsequently chosen as the standard bracketing concentration to construct concentration response curves. Two initial 100 µM ATP applications served to reduce variation in amplitude of subsequent responses and the amplitude of subsequent test concentrations were expressed as a percentage of the bracketing concentration’s response (see methods).

**Figure 6:**
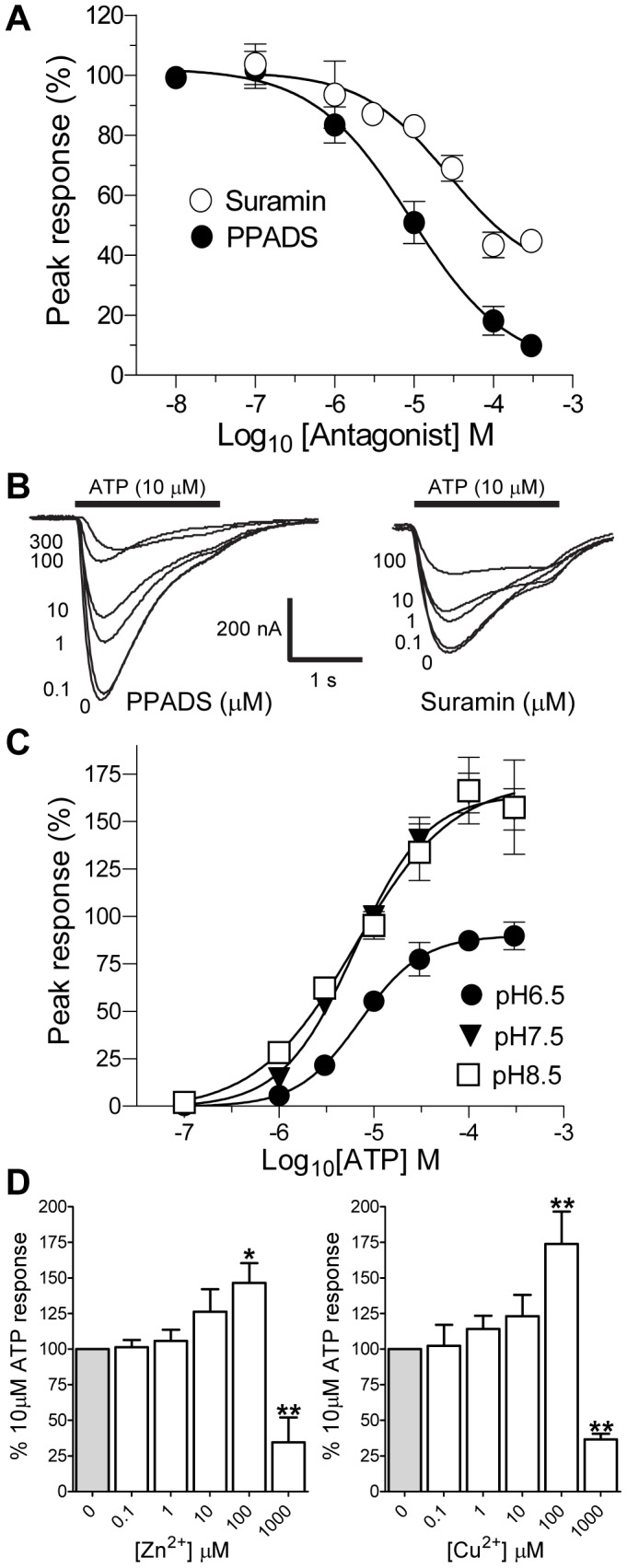
Actions of antagonists, pH and metal ions on *Lym*P2X currents. The effects of the P2 receptor antagonists suramin and PPADS, pH and zinc and copper on ATP evoked *Lym*P2X currents were determined in *Xenopus* oocytes. (A) Inhibition curves for mean responses to 10 µM ATP in the presence of suramin (open circles) and PPADS (closed circles) (n = 5−9 oocytes). Mean peak currents (± s.e.m) are normalized to responses evoked by 10 µM ATP in the absence of antagonist. (B) Representative current traces for data plotted in A) demonstrating the inhibitory effects of different concentrations of PPADS or suramin on the current response evoked by 10 µM ATP (black bar). Antagonists were bath perfused and also present in the ATP application at the appropriate concentration. (C) Concentration response curves for ATP in recording solutions of different pH. Alkaline pH 8.5 had no effect on ATP efficacy or potency whereas acidic pH 6.5 reduced current amplitudes (n = 6). Mean peak currents (± s.e.m) are normalized to responses evoked by 10 µM ATP at pH 7.5. (D) Biphasic effects of Zn^2+^ and Cu^2+^ on ATP (10 µM) evoked *Lym*P2X currents. Mean peak currents (± s.e.m) are normalized to responses evoked by 10 µM ATP in the absence of metal ion. Both Zn^2+^ and Cu^2+^ potentiated ATP evoked current amplitude when present at 100 µM (p<0.05 (*) for Zn^2+^ and p<0.01 (**) for Cu^2+^) but inhibited ATP evoked currents when present at a concentration of 1 mM (p<0.01) (n = 5−8).

Under these conditions, ATP evoked *Lym*P2X currents in a concentration-dependent manner with an EC_50_ of 6.2 µM (pEC_50_−5.2±0.07), a Hill Slope of 1.1±0.2 and a maximum response 164.3±6.2% of the 10 µM ATP-evoked response (*n* = 10) (Fig. 4A,B). The *Lym*P2X plasmid construct clone 11 was generated to incorporate the potential ATG start site at nucleotide position 3 and added the amino acid sequence MTTK to the amino terminus of the *Lym*P2X sequence. This slightly longer clone gave an EC_50_ value of 5.8 µM (pEC_50_−5.2±0.04, *n* = 5−6) for ATP (Fig. 4A) with a Hill Slope of 1.4±0.2 and a maximum response of 143.0±3.3% of the 10 µM ATP-evoked response. There was no significant difference in EC_50_ values or current characteristics between clone 11 and *Lym*P2X and the shorter clone (*Lym*P2X) was therefore chosen for all subsequent studies.

The current-voltage relationship of *Lym*P2X was investigated by recording 10 µM ATP-evoked currents under a range of holding potentials between −60 mV and +40 mV. The reversal potential was −11.4 mV (*n* = 6) and the current voltage relationship showed a slight inward rectification over the range of potentials measured (Fig. 4C and D).

**Figure 7:**
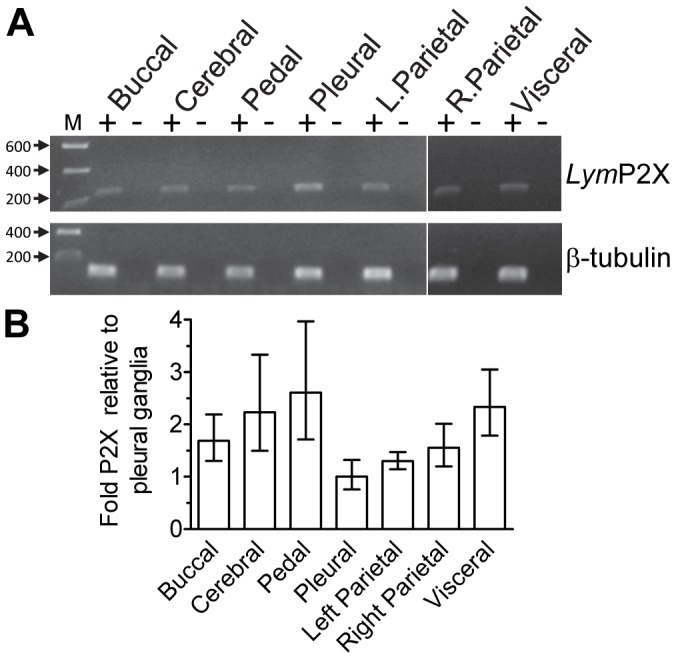
*Lym*P2X is widely expressed in *Lymnaea* CNS. (A) RT-PCR using primers for *Lym*P2X (primer pair 8 (Table 1) and β-tubulin (primer pair 9).+indicates PCR reactions on cDNA samples reverse transcribed from RNA prepared from the various ganglia indicated. – indicates negative control reactions where reverse transcriptase had been omitted from the cDNA synthesis reaction to control for genomic DNA contamination. M = molecular mass ladder (size in kb). (B) Quantitative PCR data showing the relative expression levels of *Lym*P2X in various ganglia (normalised to pleural ganglia), n = 3 independent reactions for each sample.

### Pharmacological Properties of *Lym*P2X

The ATP analogue BzATP (2′, 3′-O-(4-Benzoylbenzoyl ATP)) also evoked inward currents at *Lym*P2X with an EC_50_ of 2.4 µM (pEC_50_−5.6±0.06, *n* = 5−10) and a Hill slope of 1.1±0.2 (Fig. 5A and B). The maximal amplitude of the BzATP concentration response curve however was 33±6% smaller than that of ATP (Fig. 5A). The time course of BzATP evoked currents (10–90% rise time = 301.2±33.8 ms, T_50_ = 1.0±0.07 s, τ_desen_ = 1.2±0.09 s) was not significantly different from ATP evoked currents. αβmeATP (α,β-methyladenosine 5′-triphosphate) was a weak agonist at *Lym*P2X with 100 µM αβmeATP evoking 36.9±10.6% of the response evoked by 10 µM ATP (n = 6). It was therefore not practical to construct a concentration response curve for this agonist. Both ADP treated with hexokinase to remove ATP contamination [34] and UTP failed to evoke currents at *Lym*P2X (tested at 100 µM ) (Fig. 5B).

The general P2 receptor antagonists PPADS (pyridoxalphosphate-6-azophenyl-2′,5′-disulphonic acid) and suramin both reduced ATP evoked *Lym*P2X responses in a concentration dependent manner with IC_50_ values of 8.1 µM (pIC_50_: −5.1±0.1, Hill slope −0.7±0.1, (n = 5−9)) and 27.4 µM (pIC_50_: −4.6±0.1, Hill slope −0.8±0.1, (n = 5−6)) respectively. There was however a suramin resistant component to the *Lym*P2X current (∼40% of the maximum current) that persisted at concentrations of suramin up to 300 µM (Fig. 6A).

As pH has previously been shown to affect current amplitude in mammalian P2X receptors [35], we also tested whether *Lym*P2X can be modulated by protons by comparing ATP evoked responses in extracellular ND96 solution at pH 6.5, pH7.5 and pH 8.5. Increasing the proton concentration of the standard pH 7.5 solution 10 fold to pH to 6.5 resulted in a significant (p<0.05) decrease in current amplitudes such that 10 µM ATP-evoked responses at pH 6.5 were 55.4±4.5% of the equivalent response at pH 7.5 (Fig. 6C). The potency of ATP at pH 6.5 however was not significantly different with an EC_50_ of 7.1 (pEC_50_ −5.2±0.01, *n* = 6) and a Hill slope of 1.3±0.04. Decreasing proton concentration 10-fold from pH 7.5 to pH 8.5 had no significant effect on current amplitudes and the potency of ATP was not significantly altered (EC_50_ = 6.2 µM, pEC_50_ −5.2±0.07, Hill slope = 1.1±0.2 (n = 5–8)).

The macrocyclic lactone ivermectin is known to potentiate ATP-evoked currents at human P2X_4_ [36] and *Schistosoma mansoni* P2X receptors [10]. We therefore studied the effects of this allosteric modulator on the *Lym*P2X currents. However, ivermectin had no effect on *Lym*P2X currents (tested up to 10 µM, (n = 6), data not shown).

### Biphasic Effect of Divalent Cations at *Lym*P2X

Divalent metal cations have also previously been shown to modulate ATP-evoked currents in both vertebrate and invertebrate P2X receptors [37], [38], we therefore investigated the effects of zinc and copper on *Lym*P2X receptor function. Both Zn^2+^ and Cu^2+^displayed a biphasic effect on *Lym*P2X (Fig. 6D). An increase in Zn^2+^ or Cu^2+^ up to a concentration of 100 µM resulted in a potentiation of ATP evoked currents with responses in the presence of 100 µM metal ion being 46.5±13.9% (p<0.05) and 73.8±22.8% (p<0.01) greater than control responses respectively (n = 5–8). In contrast, responses to 10 µM ATP were significantly (p<0.01) reduced by 66.4±1.8% in the presence of 1 mM Zn^2+^ and by 63.4±4.1% in the presence of 1 mM Cu^2+^.

**Figure 8:**
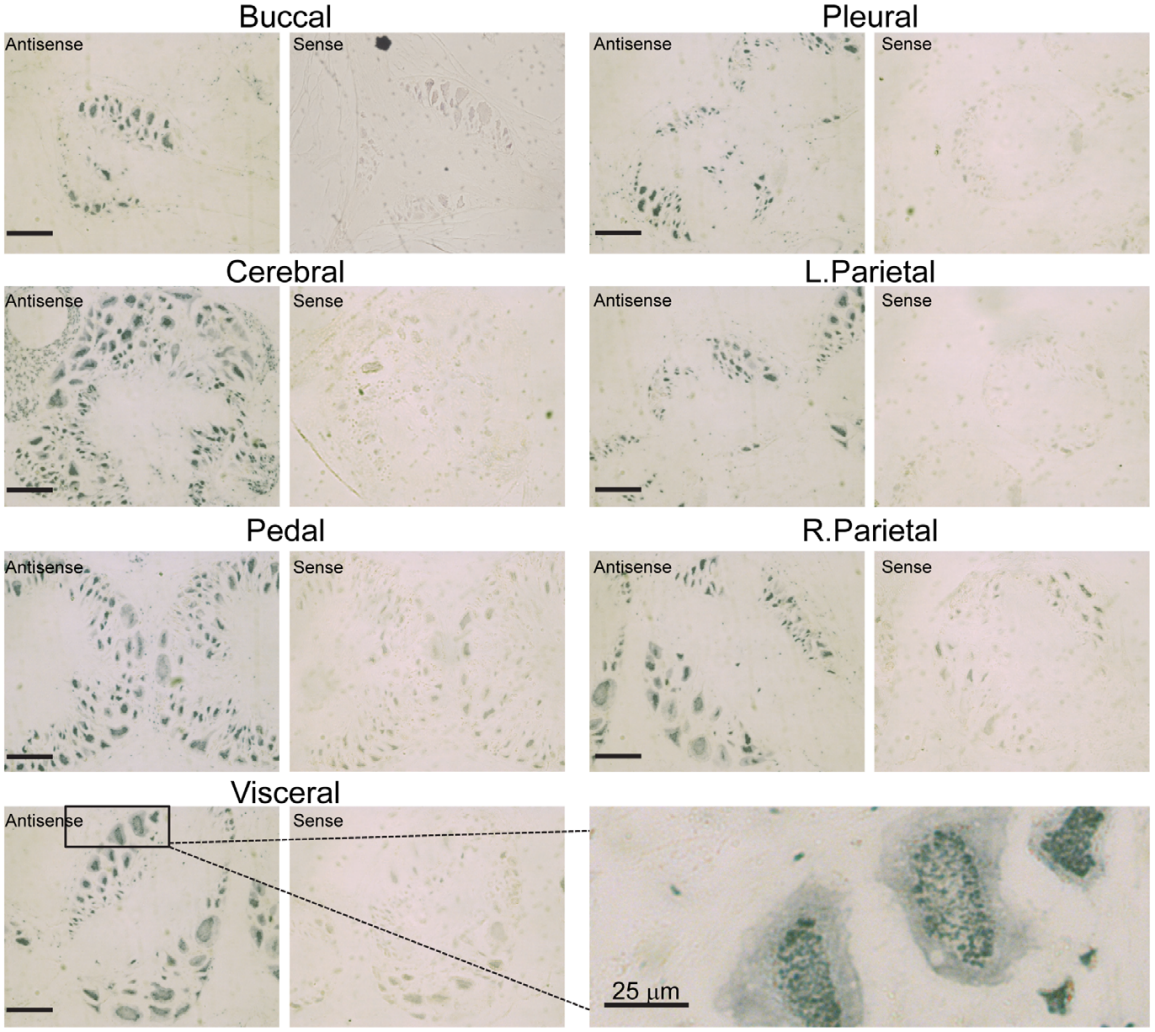
*In situ* hybridization. Wax embedded sections of *Lymnaea* CNS were probed with Digoxigenin labelled *Lym*P2X sense and antisense cRNA probes. Representative images of each ganglia are shown. Scale bars = 100 µm unless indicated otherwise. Bottom right hand panel shows a higher magnification image (scale bar = 25 µm) of neurones in the visceral ganglia, emphasising the diffuse cytoplasmic and punctate nuclear staining observed with the antisense *Lym*P2X probe.

### RT-PCR Analysis of *Lym*P2X Expression in CNS Ganglia

*Lym*P2X PCR products of the expected size (229 bp) were amplified by conventional RT-PCR in each of the 7 CNS ganglia analysed (Fig. 7A). Absence of an amplification product in the no reverse transcriptase negative control samples confirmed amplifications were from cDNA rather than genomic DNA contamination. Primers specific for β-tubulin (107 bp amplicon) served as a positive control in each ganglia RNA sample. In order to determine whether the expression level of *Lym*P2X varied between ganglia, quantitative RT-PCR using the ΔΔCt method was subsequently carried out (see methods). Pleural ganglia was found to have the least amount of *Lym*P2X expression per ng of input RNA and the ΔC_t_ of pleural ganglia was therefore selected as the ΔC_tZ_ value to be subtracted from ΔC_t_ values of other CNS ganglia. Thus *Lym*P2X expression for each ganglia sample was expressed relative to pleural ganglia (Fig. 7B). Pedal ganglia showed the highest expression of *Lym*P2X with 2.60 times (range 1.71–3.97) more *Lym*P2X RNA than the pleural ganglia. Cerebral and visceral ganglia also have over twice the quantity of *Lym*P2X mRNA than pleural ganglia.

### *In situ* Hybridization

Having determined widespread *Lym*P2X expression throughout *Lymnaea* CNS ganglia by RT-PCR and quantitative RT-PCR, we next attempted to identify individual neurones expressing *Lym*P2X by *in situ* hybridization with digoxigenin-labelled cRNA probes on paraffin-embedded sections. The *Lym*P2X antisense probe produced a strong punctate signal concentrated within the nucleus, with a weaker diffuse staining present throughout the cytoplasm (Fig. 8). This hybridization pattern was observed in the vast majority of neurons within each ganglia, with smaller neurons showing a slightly denser punctate nuclear staining than larger neurons. This strong staining pattern of the *Lym*P2X antisense probe was in marked contrast to the control sense probe (Fig. 8).

## Discussion

In this study we describe the cloning and functional characterization of a P2X receptor expressed in the CNS of the model organism *Lymnaea stagnalis*. The nested CODEHOP PCR [30] technique employed allowed us to identify a P2X receptor for which no prior gene specific EST or genomic sequence data was available. CODEHOP PCR may therefore provide a useful tool in phylogenetic studies to identify unknown P2X receptors from other organisms. By using a range of different P2X CODEHOP primer combinations, we had initially expected to amplify PCR products from several different *Lymnaea* P2X genes since P2X gene families are present in mammals which have seven genes, zebrafish with nine genes [8] and *Dictyostelium* with five genes [11], [12]. However, a single P2X gene was identified by CODEHOP PCR making it unclear whether a P2X gene family is also present in *Lymnaea*. Similarly, from BLAST searches of 116,355 sequence contigs produced by a recent Deep RNA sequencing transcriptome analysis of *Lymnaea* CNS [39], we identified a single P2X encoding contig, rather than a family of P2X genes. This contig (Accession FX193730) appears to represent the same gene as *Lym*P2X. However, a region of 150 nucleotides which encode 50 amino acids within the P2X cysteine rich head domain differ markedly between *Lym*P2X and FX19370 suggesting the presence of an alternatively spliced exon within this gene. BLAST searches of complete genome data for the limpet *Lottia gigantean* (Department of Energy Joint Genome Institute) and >175,000 EST sequences from the sea slug *Aplysia californica* [40] also identified single P2X genes in each of these species rather than a family of genes. Thus, a lineage-specific gene expansion similar to those which led to the independent emergence of P2X gene families in vertebrates and *Dictyostelium* [17] may not have occurred in gastropods. Indeed the wide range of organisms for which just a single P2X gene has been identified [1], [14], [41], [42] and the fact that several model organisms with full genome data lack P2X receptors [10], [17] suggests that the maintenance, or loss, of a single ancestral P2X gene may have been the norm over eukaryotic evolution rather than expansion into a P2X gene family.

Heterologous expression of *Lym*P2X in *Xenopus* oocytes allowed us to record ATP evoked inward membrane currents and therefore provide definitive functional evidence for the existence of P2X receptors in the phylum mollusca. The pharmacological data gathered in this study will be useful in future studies to identify the *in vivo* roles of *Lym*P2X in relation to potential P2Y receptors that could also be present in this organism. In comparison to the human P2X1-7 subtypes, *Lym*P2X shows most amino acid sequence identity to P2X4. ATP evoked currents at *Lym*P2X were also P2X4-like in that they displayed relatively slow current kinetics, low sensitivity to αβmeATP and an EC_50_ of 6.2 µM for ATP. However, unlike P2X4 *Lym*P2X is insensitive to ivermectin and is antagonised by both PPADS and suramin (Fig. 6A). Several predominantly nonpolar residues present in both transmembrane domains (highlighted in Fig. 2) have been shown to be involved in the actions of ivermectin at rat P2X4 receptors (reviewed in [6]). The pattern of these residues is consistent with the helical topology of the transmembrane domains [6] and is largely conserved in the *Schistosoma mansoni* and *Hypsibius dujardini* P2X receptors which are also sensitive to ivermectin (Fig. 2). The rat P2X4 TM2 domain ivermectin sensitive residues N338 G342, L346 A349 and I356 are completely conserved in *Lym*P2X suggesting that the reason for the insensitivity of *Lym*P2X to ivermectin could lie within the TM1 domain where two of the four identified rat P2X4 ivermectin sensitive residues in *Lym*P2X differ from the other ivermectin sensitive receptors (Fig. 2).

Similar to P2X_1_, P2X_3_ and P2X_4_ [35], [36], [43], *Lym*P2X currents are inhibited by acidic pH with a reduced amplitude and maximal response at pH 6.5 compared to pH 7.5 (Fig. 6C). This modulation of *Lym*P2X function by acidic pH could be of physiological significance since acidic shifts are known to occur at the synaptic cleft during the release of neurotransmitters from pre-synaptic vesicles [44], [45] and localised acidosis also occurs during tissue injury, ischaemia and inflammation [44] which could also potentially modulate P2X receptor function.

The modulation of P2X receptor function by divalent metal ions is also of physiological significance since zinc is present in presynaptic vesicles and is released into the synaptic cleft on nerve stimulation [46]–[48]. Zinc has also been shown to co-localise with P2X receptors in the hippocampus and cerebral cortex, implying a possible modulatory role [48], [49]. The biphasic action of zinc and copper on *Lym*P2X currents (Fig. 6C) implies that the receptor has two distinct sites of modulation susceptible to divalent cations; a high affinity site leading to potentiation, and following saturation of this high affinity site, a lower affinity site, leading to inhibition. Biphasic effects of zinc have also been described *in vivo* in P2X expressing hypothalamic tuberomamillary neurons in rat [50]. Similarly, both zinc and copper also have biphasic effects on the dorsal motor nucleus of the rat vagus nerve [51]. None of the residues thought to be involved in the formation of the metal ion binding sites in rat P2X2 [52]–[54], human P2X2 [55] or rat P2X4 [56] are conserved in *Lym*P2X (Fig. 2), further highlighting the fact that, unlike the ATP binding site, there seems to be little conservation of residues involved in the coordination of metal ion binding between P2X receptor subtypes [6].

*Lym*P2X was found to be expressed in all *Lymnaea* CNS ganglia by both conventional and quantitative RT-PCR (Fig. 7). *Lym*P2X mRNA was most abundant in pedal ganglia, where expression was between 1.7 to 4 times greater than pleural ganglia. Consistent with this RT-PCR data, *in situ* hybridization (Fig. 8) also revealed expression of *Lym*P2X mRNA in the majority of neurons in each CNS ganglia. The *Lym*P2X antisense probe hybridized predominantly to the nucleus of neurons, displaying a strong punctate staining pattern. This nuclear binding is unlikely due to hybridization to genomic DNA since the control sense probe did not show a strong punctuate staining pattern and each nucleus contained multiple puncta. A more diffuse pattern of *Lym*P2X antisense cRNA staining was also observed in the cytoplasm of positive cells demonstrating that *Lym*P2X mRNA was not restricted to the nucleus. A predominantly nuclear localization of mRNA has also previously been reported for other genes including Cytochrome P450 1B1 in human neurons and astrocytes [57] and the transcription factor WT1 which has been implicated in tumorigenesis [58]. The presence of *Lym*P2X mRNA at a similar level in the majority of neurones in all CNS ganglia implies a general role of the *Lym*P2X channel in CNS function rather than a specific role in a particular neuronal pathway. Indeed, this widespread localisation of *Lym*P2X is consistent with the similarly widespread release of ATP from all *Lymnaea* ganglia upon addition of neuroexcitatory compounds [29] suggesting that purinergic synaptic transmission could be widely utilised in *Lymnaea* CNS.

In summary, this study has established the existence of a P2X receptor in the CNS of the mollusc *Lymnaea stagnalis* and determined the functional properties of this channel by heterologous expression in *Xenopus* oocytes. The comparative simplicity of the *Lymnaea* CNS with large readily identifiable neurones makes this organism an attractive model system and the pharmacological data obtained in this study will be valuable in future *in vivo* studies to probe the physiological roles of *Lym*P2X in *Lymnaea* CNS function.
